# New Brazilian Cerambycidae from the Amazonian region (Coleoptera)

**DOI:** 10.3897/zookeys.603.7335

**Published:** 2016-07-06

**Authors:** Antonio Santos-Silva, Maria Helena M. Galileo

**Affiliations:** 1 Museu de Zoologia, Universidade de São Paulo, São Paulo, SP, Brazil; 2 PPG Biologia Animal, Departamento de Zoologia, Universidade Federal do Rio Grande do Sul, Porto Alegre, RS, Brazil. (Fellow of the Conselho Nacional de Desenvolvimento Científico e Tecnológico)

**Keywords:** Neotropical region, taxonomy

## Abstract

Three new species of Cerambycidae are described from the Brazilian Amazonian region: *Psapharochrus bezarki* (Lamiinae, Acanthoderini); *Xenofrea ayri* (Lamiinae, Xenofreini); and *Mecometopus wappesi* (Cerambycinae, Clytini). *Mecometopus wappesi* is added to a previous key.

## Introduction

*Psapharochrus* Thomson, 1864 is a large genus of Acanthoderini from the American continent, currently with 92 species. Monné (2015b) recorded 90 species, not including *Psapharochrus wappesi* Galileo et al., 2015, and *Psapharochrus langeri* Martins et al., 2015. However, this catalogue includes *Psapharochrus quadrigibbus* (Say, 1831), a species currently belonging to Acanthoderes (Acanthoderes) Audinet-Serville, 1835 (e.g. Audureau 2010), and does not include *Psapharochrus lengii* (Wickhan, 1914), from the U.S.A, a species described in *Acanthoderes* and transferred to *Psapharochrus* by Linsley (1961), without explanation. Twenty one species are currently recorded from the Brazilian Amazonian region.

*Xenofrea* Bates, 1885 is an exclusively American genus, occurring from Mexico (Chiapas) to Central and South America. According to Monné (2015b), the genus encompasses 52 species. With the recent description of *Xenofrea wappesi* Galileo et al., 2015, currently *Xenofrea* includes 53 species. Sixteen species are recorded from the Brazilian Amazonian region.

*Mecometopus* Thomson, 1861 encompasses 14 species, occurring from Mexico to Southern South America (Monné 2015a). Currently eight species are recorded from the Brazilian Amazonian region.

## Material and methods

Photographs were taken with a Canon EOS Rebel T3i DSLR camera, Canon MP-E 65mm f/2.8 1–5× macro lens, controlled by Zerene Stacker AutoMontage software. Measurements were taken in ‘‘mm’’ using a micrometer ocular Hensoldt/Wetzlar - Mess 10 in the Leica MZ6 stereomicroscope, also used in the study of the specimens.

The collection acronyms used in this study are as follows:

INPA
 Coleção Sistemática de Entomologia, Instituto Nacional de Pesquisas da Amazônia, Manaus, Amazonas, Brazil 


MZSP
 Museu de Zoologia, Universidade de São Paulo, São Paulo, Brazil 


## Systematics

### Acanthoderini Thomson, 1860

#### Psapharochrus bezarki sp. n.

Taxon classificationAnimaliaColeopteraCerambycidae

http://zoobank.org/C131C15F-145C-4412-B6DA-4A1BB126D4CA

Figs 1–5

##### Description.

Holotype female. Integument dark-brown; mouthparts reddish-brown, except for palpi mostly dark-brown.

*Head*. Frons moderately coarsely, sparsely punctate; with ochraceous pubescence on wide band on each side of longitudinal sulcus, longitudinal lateral wide band connected to transverse band below antennal tubercles and narrow band around eyes; remaining surface glabrous or nearly so. Area between antennal tubercles moderately, coarsely punctate laterally, smooth centrally; with ochraceous pubescence close to antennal tubercles, glabrous centrally. Vertex moderately, coarsely punctate between upper eye lobes, impunctate on remaining surface; on each side with large, elliptical macula with brown pubescence, surrounded laterally and posteriorly with dense, yellowish pubescence (becoming wider behind upper eye lobe); remaining surface with slightly conspicuous brownish pubescence. Area behind eyes microsculptured on wide band close to eye, moderately, finely, abundantly punctate on remaining surface; glabrous, except for narrow pubescent band close to eye. Genae transversely striate laterally, very finely striate and punctate toward frons; with short, ochraceous, sparse setae, except for narrow band close to eye. Submentum with transverse, narrow central carina; microsculptured, with short, ochraceous pubescence. Antennal tubercles mostly glabrous, impunctate. Longitudinal sulcus distinct from clypeus to anterior margin of prothorax. Distance between upper eye lobes 0.55 times length of scape; distance between lower eye lobes in front equal to length of scape. Antennae 1.4 times elytral length; reaching elytral apex; scape with brownish pubescence, maculate with ochraceous pubescence; antennomeres III–IV with yellowish-white pubescence on base and transverse band before apex, remaining surface with brownish pubescence; antennomeres V–X with yellowish-white pubescence on basal third, brown on distal third (gradually widening toward X); antennomere XI with yellowish-white pubescence; antennal formula based on antennomere III: scape = 0.70; pedicel = 0.18; IV = 0.71; V = 0.51; VI = 0.41; VII = 0.37; VIII = 0.32; IX = 0.31; X = 0.26; XI = 0.26.

*Thorax*. Prothorax 1.8 times wider than long (including lateral tubercles); lateral tubercles, large, conical, with blunt apex. Pronotum coarsely, deeply, sparsely punctate, finer, denser on each side of central tubercle; with three distinct tubercles: one on each side, very large, reniform; another centrally, triangular at base, carina-shaped toward apex; with ochraceous pubescence, denser on some regions, absent or nearly so on others. Sides of prothorax coarsely, moderately abundantly punctate; with yellowish-white, dense pubescence, less so near anterior margin. Prosternum impunctate; with yellowish-white pubescence laterally and close to coxal cavities. Prosternal process wide, centrally distinctly wider than base of peduncle of profemora; with yellowish-white pubescence, not obscuring integument. Mesosternum microsculptured, except for smooth, transverse anterior band; with short, moderately sparse, ochraceous pubescence, but glabrous on smooth region. Mesepisterna, mesepimera, and metepisterna with dense yellowish-white pubescence. Metasternum with dense yellowish-white pubescence; centrally rubbed in the holotype. Scutellum centrally depressed distally; distal lateral sides distinctly elevated; with brown pubescence, except for narrow yellowish band at apex.

*Elytra*. Sides slightly convergent toward distal third, then gradually curved toward apex; with distinct tubercle on each side of scutellum; without distinct carinae; coarsely, sparsely punctate; central area of disc on basal third mostly glabrous; remaining surface of basal 2/3 with ochraceous pubescence, mixed with brown and white pubescence (laterally, on center of this region, with distinct, oblique band of brown pubescence); with zig-zag, transverse band of brown pubescence about beginning of distal third (not reaching suture); with transverse band of brown pubescence on distal quarter; along suture, with rounded spots of brown pubescence; remaining surface of distal third with ochraceous pubescence mixed with brown and white pubescence; apex truncate, with outer angle slightly projected and sutural angle rounded.

*Legs*. Femora and tibiae with yellowish-white pubescence, except for golden pubescence on dorsal sulcus of mesotibiae and ventral apex of meso- and metatibiae.

*Abdomen*. Ventrites microsculptured; with yellowish-white pubescence (partially rubbed in the holotype); ventrite V with longitudinal, narrow, central sulcus on basal 3/4.

**Figures 1–7.**
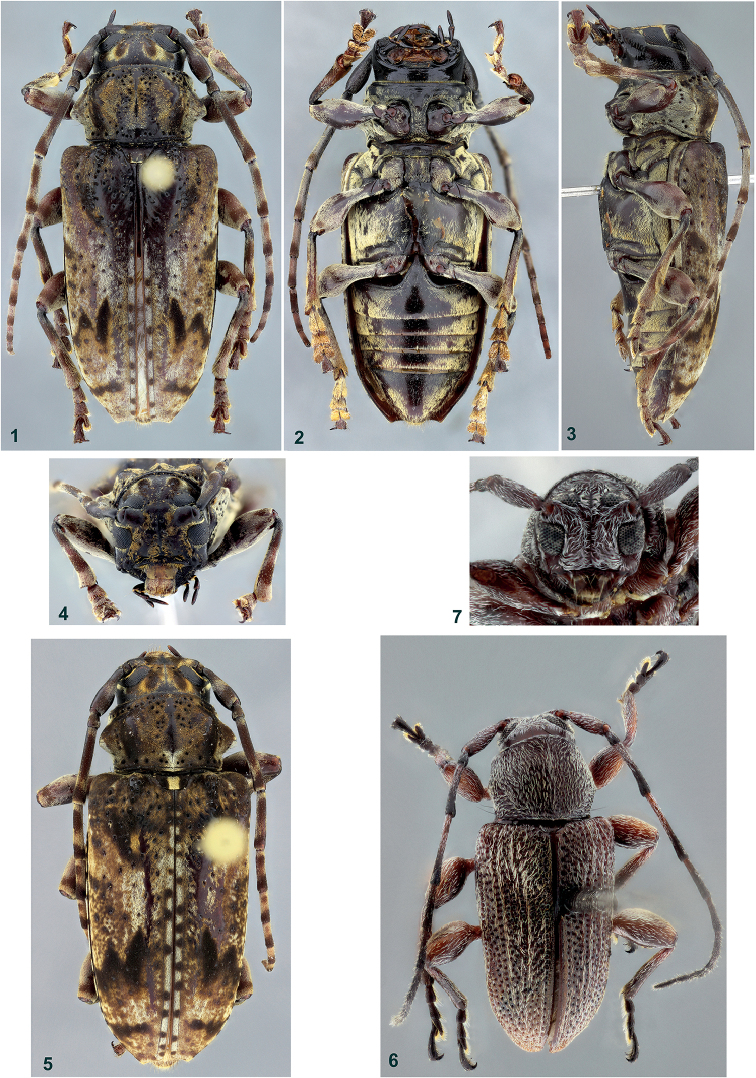
**1–5** *Psapharochrus bezarki*, female: **1** dorsal habitus, holotype **2** ventral habitus, holotype **3** lateral habitus, holotype **4** head, frontal view, holotype **5** dorsal habitus, paratype **6–7** *Xenofrea ayri*, holotype male: **6** dorsal habitus **7** head, frontal view.

##### Variation.

Frons totally pubescent, but with glabrous area on holotype covered with short, yellowish-brown pubescence; pubescence on frons ochraceous, almost covering entire surface (except for elliptical brown macula); antennal tubercles mostly with ochraceous pubescence; scutellum centrally with wide, yellowish band of pubescence; elytral without glabrous area on basal third; pubescence on ventral side of body more ochraceous.

##### Dimensions in mm

**(female)**. Total length (from mandibular apex to abdominal apex), 16.4–17.8; prothorax: length, 2.9–3.4; anterior width, 4.3–4.8; posterior width, 4.3–4.8; largest width, 5.4–6.1; humeral width, 6.3–6.9; elytral length, 11.5–12.7. The largest dimensions are those of the holotype.

##### Type material.

Holotype female from BRAZIL, *Amazonas*: Manaus (ZF2, km 14, Torre – 40 m high, 02°35'21"S / 60°06'55"W, light trap), 19-22.III.2004, J. A. Rafael, C. S. Motta, F. F. Xavier Filho, A. Silva Filho and J. T. Câmara col. (INPA). Paratypes - BRAZIL, *Amazonas*: Manaus (ZF2, km 14, Torre – 35 m high, 02°35'21"S / 60°06'55"W, light trap), female, 13-16.VIII.2004, J. A. Rafael, F. F. Xavier Filho, A. R. Ururahy, A. Silva Filho and S. Trovisco col. (MZSP); female, 9-12.XI.2004, C. S. Motta, A. S. Filho, S. Trovisco and L. S. Aquino col. (INPA).

##### Etymology.

The new species is named after Larry G. Bezark, for his contribution toward the knowledge of Cerambycidae, his friendship, and constant help.

##### Remarks.

*Psapharochrus bezarki* sp. n. is similar to *Psapharochrus bimaculatus* (Fuchs, 1959), but differs by the elytra more parallel-sided (distinctly more narrowed toward apex in *Psapharochrus bimaculatus*), and by the presence of the zig-zag brown macula on the distal half of the elytra (absent in *Psapharochrus bimaculatus*). It differs from *Psapharochrus nigropunctatus* (Tippmann, 1960) by the scutellum proportionally larger, longitudinally sulcate posteriorly (smaller and flat in *Psapharochrus nigropunctatus*), and by the protibiae not laterally flattened (flattened in *Psapharochrus nigropunctatus*). It can be separated from *Psapharochrus lanei* (Marinoni & Martins, 1978) by the lateral tubercles of pronotum reniform (subconical in *Psapharochrus lanei*), by the scutellum larger and longitudinally sulcate posteriorly (smaller and flat in *Psapharochrus lanei*), and by the lateral tubercles of prothorax with blunt apex (acute in *Psapharochrus lanei*).

### Xenofreini Aurivillius, 1923

#### Xenofrea ayri sp. n.

Taxon classificationAnimaliaColeopteraCerambycidae

http://zoobank.org/84B64850-D902-4005-A1C9-F24B9D0CEBCA

Figs 6–7 , 8–9

##### Description.

Holotype male. Integument dark-brown, almost black; base of antennomeres III–V, coxae, femora, and most tibiae dark reddish-brown; abdominal ventrites brown.

*Head*. Frons, area between eyes and vertex finely, abundantly punctate; with gray pubescence, not entirely obscuring integument. Area behind eyes microsculptured, mainly toward lower lobe; with wide band of gray pubescence close to eye, glabrous toward prothoracic margin. Genae finely, abundantly punctate close to eye, smooth on apex; with gray, moderately sparse pubescence. Antennal tubercles covered with gray pubescence. Longitudinal sulcus distinct from clypeus to posterior margin of upper eye lobes. Distance between upper eye lobes 0.3 times length of scape; distance between lower eye lobes in frontal view 0.6 times length of scape. Antennae 1.85 times elytral length; reaching elytral apex at middle of antennomere VIII; antennomeres with short, gray pubescence interspersed with short, erect yellowish setae (denser toward distal antennomeres); antennal formula based on antennomere III: scape = 0.91; pedicel = 0.27; IV = 1.24; V = 0.72; VI = 0.69; VII = 0.67; VIII = 0.57; IX = 0.51; X = 0.48; XI = 0.51.

*Thorax*. Prothorax 1.3 times wider than long (including lateral tubercles); lateral tubercles placed before middle, blunt; anterolateral tubercles slightly distinct. Pronotum finely, abundantly punctate; with moderately thick, decumbent, abundant, gray setae, distinctly not obscuring integument, slightly denser laterally and on narrow, longitudinal, central band on anterior half; basal margin straight; anterior margin, rounded, somewhat projected forward centrally. Sides of prothorax with sculpture and setae as on pronotum (punctures slightly coarser). Prosternum notably narrow, about 1/3 of length of procoxal cavity; finely, densely punctate, with very short, decumbent setae. Prosternal process centrally narrowed, narrowest area as wide as half of base of peduncle of profemora. Mesosternum about as long as prosternum; finely, densely punctate; with short, decumbent, gray setae, not obscuring integument. Mesepisterna and mesepimera finely, abundantly punctate (punctures slightly coarser than on mesosternum); with gray, decumbent setae (longer than on mesosternum), not obscuring integument. Metepisterna with gray, decumbent, dense setae, obscuring integument. Metasternum with gray, dense pubescence. Scutellum with gray, moderately sparse, decumbent setae.

*Elytra*. Sides slightly convergent from humerus to about distal third, then rounded, narrowed toward sutural angle; coarsely, densely punctate (slightly finer toward apex); with gray, thick, short setae forming longitudinal rows (somewhat less distinctly on anterior quarter).

*Legs*. Femora notably clavate; with decumbent, gray pubescence, not obscuring integument. Tibiae mostly with gray, decumbent, short setae.

*Abdomen*. Ventrites finely, abundantly punctate; with decumbent, grayish setae, not obscuring integument.

##### Dimensions in mm.

Total length, 4.40; prothorax: length, 1.00; anterior width, 1.15; posterior width, 1.15; largest width, 1.40; humeral width, 1.75; elytral length, 3.15.

**Figures 8–13.**
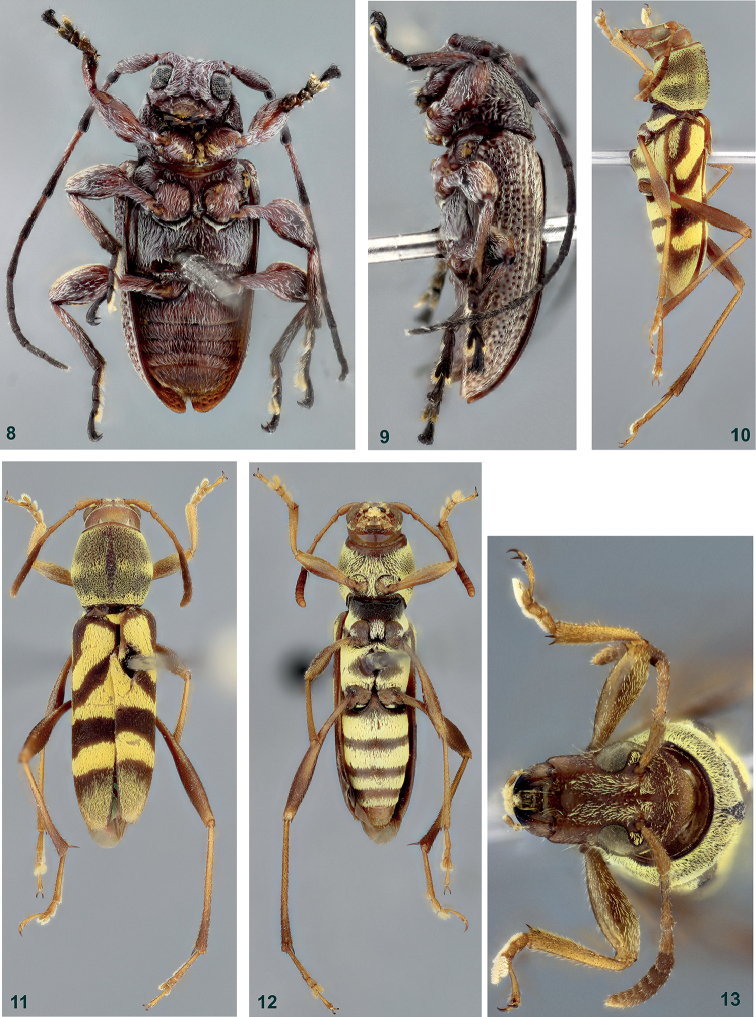
**8–9** *Xenofrea ayri*, holotype male: **8** ventral habitus **9** lateral habitus **10–13** *Mecometopus wappesi*, holotype male: **10** lateral habitus **11** dorsal habitus **12** ventral habitus **13** head, frontal view.

##### Type material.

Holotype male from BRAZIL, *Amazonas*: Novo Airão (02°38'39“S / 60°56'07"W; armadilha luminosa, dossel, 18:00–21:00h), 27.VIII.2011, F. F. Xavier & A. Agudelo col. (INPA).

##### Etymology.

Tupi, ayrí = tiny; relating to the small size of the species.

##### Remarks.

*Xenofrea ayri* sp. n. is the smallest known species of the genus, and can be easily recognized by the elytral pubescence forming rows, while in the other species the elytra always have different complex patterns.

### Clytini Mulsant, 1839

#### Mecometopus wappesi sp. n.

Taxon classificationAnimaliaColeopteraCerambycidae

http://zoobank.org/C8FCF792-A63C-44D3-AF05-13340F85BD33

Figs 10–13

##### Description.

Holotype male. Head reddish-brown, more brownish on some areas; mandibles reddish-brown on basal 2/3, dark-brown on apical third; scape, pedicel and antennomeres III–IV reddish-brown; antennomeres V–XI brown, more reddish ventrally on distal antennomeres; prothorax dark-brown, except for reddish-brown anterior region of prosternum; mesosternum dark-brown; remaining ventral surface reddish-brown; elytra dark-brown, except for reddish-brown areas under dense yellow pubescence; pro- and mesofemora mostly reddish-brown (slightly darker on mesofemora), except for yellowish-brown distal area of club; metafemora mostly brown, except for reddish brown region of peduncle and part of club, and apex of club; pro- and mesotibiae yellowish-brown; metatibiae yellowish-brown on basal 2/3, reddish-brown on apical third; tarsi from yellowish-brown to reddish-brown. Pubescence mostly yellow, more whitish on mesosternal process, and yellowish-white on ventral side of meso- and metathorax and abdomen; brown on dark regions of elytra.

*Head*. Frons finely, densely punctate, except for narrow, longitudinal, central band and triangular area close to clypeus with punctures slightly coarser, distinctly sparser; with wide band of short, decumbent setae on each side (distinctly not obscuring integument). Area between antennal tubercles and upper eye lobes with sculpture and setae as on frons. Vertex microsculptured interspersed with fine, moderately sparse punctures, except for narrow, smooth, longitudinal, central band; with very short, sparse setae, except for longer, denser setae close to basal area. Area behind eyes finely, abundantly punctate (punctures slightly coarser, sparser toward margin of prothorax); with moderately dense, narrow band of pubescence close to eyes (wider toward apex of upper eye lobe); remaining surface glabrous. Area between gena and submentum with long, sparse setae. Genae 1.3 times as long as lower eye lobe; finely, abundantly punctate, except for smooth, narrow area close to apex; with short, sparse setae (sparser toward apex). Submentum smooth, except for some small, very sparse asperites; with short, moderately sparse setae (slightly denser laterally) interspersed with long setae. Antennal tubercles with sculpture and setae as on frons, except for narrow glabrous, smooth area close to apex. Distance between upper eye lobes 1.15 times length of scape; distance between lower eye lobes in frontal view 0.95 times length of scape. Antennae 0.7 times elytral length; reaching about basal quarter of elytra; antennal formula based on antennomere III: scape = 1.03; pedicel = 0.42; IV = 0.64; V = 0.67; VI = 0.53; VII = 0.39; VIII = 0.32; IX = 0.28; X = 0.21; XI = 0.25.

*Thorax*. Prothorax as long as wide at widest region; sides rounded. Pronotum coarsely, densely punctate; longitudinal carina distinct from basal quarter to near anterior margin, enlarged at middle, with small transverse oblique keels; with three wide, transverse bands of dense pubescence, fused on lateral side of prothorax: one basally, narrowed on middle; one centrally, interrupted by the longitudinal carina; one close to anterior margin; remaining surface with pubescence sparser, mainly on longitudinal carina. Sides of prothorax with sculpture and pubescence as on pronotum. Prosternum with sculpture as on pronotum, except for transverse band at anterior quarter finely striate and punctate; pubescence dense, obscuring integument, except for subglabrous, transverse band at anterior quarter. Prosternal process moderately narrowed centrally; distal half deeply, widely sulcate centrally; with dense pubescence. Mesosternum finely rugose; with short, sparse setae, except for small region with dense pubescence close to mesocoxal cavities and mesepisterna. Mesepisterna with sparse pubescence on anterior region, notably dense on posterior region. Mesepimera with brown, sparse pubescence. Mesosternal process with dense pubescence. Scutellum densely yellow pubescent. Metepisterna with dense, yellow pubescence, except for narrow anterior band with brown, sparse pubescence. Metasternum densely pubescent, except for transverse, wide band with distinctly sparser pubescence. Elytra. Each elytron with five wide areas with dense, yellow pubescence: one at basal third, oblique, distinctly enlarged from side to anterior margin (not reaching lateral and anterior margin); one longitudinal laterally at basal quarter; one before middle, triangular, narrowed toward side, then projected forward (reaching suture, almost reaching lateral margin); one transverse, about middle of distal half (reaching suture, almost reaching lateral margin); one covering almost entire distal quarter, less dense. Elytral apex obliquely truncate, with small spine at outer and sutural angles.

*Legs.* Inner and outer apex of metafemora triangularly projected.

*Abdomen*. Ventrites I–IV densely pubescent distally, distinctly sparser on anteriorly (this latter gradually wider from I to IV). Ventrite V with sparse pubescence throughout.

##### Dimensions in mm.

Total length, 8.7; prothorax: length, 2.1; anterior width, 1.5; posterior width, 1.6; largest width, 2.1; humeral width, 2.1; elytral length, 5.5.

##### Type material.

Holotype male from BRAZIL, *Amazonas*: 60 Km N Manaus (Fazenda Esteio; ZF-3 km 23), 6.XII.1984, B. C. Klein col. (INPA).

##### Etymology.

The new species is named after James E. Wappes, for his contribution toward the knowledge of Cerambycidae, friendship, and constant help.

##### Remarks.

*Mecometopus wappesi* sp. n. is similar to *Mecometopus globicollis* (Laporte & Gory, 1841), but differs as follows: body distinctly slender; pronotum covered with yellow pubescence; yellow triangular macula on the elytra reaches the sides and is then projected forward; distal quarter of the elytra with yellow pubescence. In *Mecometopus globicollis* (see photograph of the holotype at Bezark 2015) the body is wider, the pronotum is not covered with yellow pubescence, the yellow triangular macula on the elytra does not reach the sides, and the distal quarter of the elytra has no yellow pubescence.

*Mecometopus wappesi* also resembles *Miriclytus triangularis* Martins & Galileo, 2008, but differs mainly by the antennae distinctly 11-segmented (antennomeres VIII–XI fused in *Miriclytus*), by the transverse and oblique bands of yellow pubescence on the elytra being wider (narrow in *Miriclytus triangularis*).

*Mecometopus wappesi* can be included in the alternative of couplet “11”, from Martins and Galileo (2011) (translated; modified):

**Table:** 

11(10)	Sides at middle of elytra without small spot of white pubescence	**11**’
–	Sides at middle of elytra with small spot of white pubescence	**12**
11’(11)	Pronotum with distinct yellow pubescence; yellow triangular macula of elytra reaching sides; apical quarter of elytra with yellow pubescence. Brazil (Amazonas)	***Mecometopus wappesi* sp. n.**
–	Pronotum without yellow pubescence; yellow triangular macula of elytra not reaching sides; apical quarter of elytra without yellow pubescence. French Guiana, Brazil (Amazonas, Pará, Maranhão)	***Mecometopus globicollis* (Laporte & Gory, 1836)**

## Supplementary Material

XML Treatment for Psapharochrus bezarki

XML Treatment for Xenofrea ayri

XML Treatment for Mecometopus wappesi

## References

[B1] Audureau A (2010) Complément a l’inventaire faunistique des Cerambycidae de la reserve privée forestière de Domitila (Nicaragua) aves description de nouvelles espèces (Coleoptera, Cerambycidae). Les Cahiers Magellanes 110: 1–10.

[B2] Bezark LG (2016) A photographic Catalog of the Cerambycidae of the New World. https://apps2.cdfa.ca.gov/publicApps/plant/bycidDB/wsearch.asp?w=n [accessed November 2015]

[B3] Linsley EG (1961) The Cerambycidae of North America. Part I. Introduction. University of California Publications in Entomology 18: 1–97.

[B4] Martins UR Galileo MHM (2011) Tribo Clytini. In: Martins UR (Eds) Cerambycidae Sul-Americanos (Coleoptera). Taxonomia. Sociedade Brasileira de Entomologia, Curitiba, v. 12, 8–264.

[B5] Monné MA (2015a) Catalogue of the Cerambycidae (Coleoptera) of the Neotropical Region. Part I. Subfamily Cerambycinae. http://www.cerambyxcat.com/Part1_Cerambycinae.pdf [accessed November 2015]

[B6] Monné MA (2015b) Catalogue of the Cerambycidae (Coleoptera) of the Neotropical Region. Part I. Subfamily Lamiinae. http://www.cerambyxcat.com/Part2_Lamiinae.pdf [accessed November 2015]

